# Women's Representation in 60 Occupations from 1972 to 2010: More Women in High-Status Jobs, Few Women in Things-Oriented Jobs

**DOI:** 10.1371/journal.pone.0095960

**Published:** 2014-05-02

**Authors:** Richard A. Lippa, Kathleen Preston, John Penner

**Affiliations:** Department of Psychology, California State University, Fullerton, California, United States of America; State University of New York, Oswego, United States of America

## Abstract

To explore factors associated with occupational sex segregation in the United States over the past four decades, we analyzed U.S. Bureau of Labor Statistics data for the percent of women employed in 60 varied occupations from 1972 to 2010. Occupations were assessed on status, people-things orientation, and data-ideas orientation. Multilevel linear modeling (MLM) analyses showed that women increasingly entered high-status occupations from 1972 to 2010, but women's participation in things-oriented occupations (e.g., STEM fields and mechanical and construction trades) remained low and relatively stable. Occupations' data-ideas orientation was not consistently related to sex segregation. Because of women's increased participation in high-status occupations, occupational status became an increasingly weak predictor of women's participation rates in occupations, whereas occupations' people-things orientation became an increasingly strong predictor over time. These findings are discussed in relation to theories of occupational sex segregation and social policies to reduce occupational sex segregation.

## Introduction

Despite dramatic changes in gender roles in recent decades, labor markets across the world continue to show marked sex segregation [1]–[4]. Researchers have estimated that to achieve equal male and female representation in U. S. jobs, approximately 50 percent of currently employed individuals would have to be reassigned to other jobs [5]–[6]. The causes of occupational sex segregation are complex and multifaceted. Factors studied by social scientists include: the influence of gender socialization, gender roles, and gender stereotypes; social policies that make it difficult for women to easily combine work and family roles; differences in the educational backgrounds and human capital of men and women; sex differences in interests, values, motivation, and abilities; and sex-linked genetic and hormonal influences [4], [7], [8]. In recent years, researchers have focused particular attention on sex segregation in STEM (science, technology, engineering, and math) fields, which offer strong employment opportunities, good pay, and high status, but which simultaneously suffer from strong gender imbalances favoring men [7], [9], [10].

Despite its ubiquity, occupational sex segregation is not fully understood, and social scientists continue to investigate its causes and correlates. Researchers have identified several empirical puzzles in research findings on occupational sex segregation that require explanation [3]. First, although conventional wisdom holds that “the best jobs go to men,” the correlation between occupations' status and sex segregation is often weak, and today not all high-status jobs are dominated by men [11]. Second, although occupational sex segregation occurs in virtually all societies, it tends to be stronger in economically developed countries with liberal gender ideologies than in less developed countries with more traditional gender ideologies [12], [13]. This pattern is problematic for theories that appeal to patriarchy, gender roles, and gender stereotypes as causes of occupational sex segregation. Finally, despite striking changes in gender roles in recent decades and dramatic increases in the number of women in the workforce, occupational sex segregation has, in comparison, declined relatively slowly—and much more slowly for some occupations than others. Many STEM fields, for example, continue to show strong sex segregation, with women's rates of participation much lower than men's [7], [9].

In an attempt to address the complexity of empirical findings on occupational sex segregation, researchers have often distinguished between “vertical” and “horizontal” segregation [2], [11]. Vertical segregation is based on “job quality,” with men tending to work in “higher quality” (i.e., higher status and higher paying) jobs than women. In contrast, horizontal segregation operates at a given status level to assign men and women to different kinds of work based on a variety of occupational characteristics. For example, one important job dimension linked to horizontal sex segregation is a manual-nonmanual continuum, with men assigned more to manual work and women to nonmanual work [3].

There are undoubtedly other important job characteristics that contribute to sex segregation as well. Two fundamental dimensions of occupational variation that have been much studied by vocational interest and individual difference researchers are the people-things dimension and the data-ideas dimension [14]–[17]. The first dimension taps the degree to which occupations deal with people and their psychological dynamics versus inanimate things and mechanical systems. The second dimension taps the degree to which occupations entail routine record-keeping and data management versus creative thinking and the use of intelligence. While women and men do not differ much in their preference for ideas-oriented versus data-oriented jobs, they do differ substantially in their preferences for people-oriented versus things-oriented jobs, with women expressing greater preference for people-oriented jobs and men for things-oriented jobs [14], [18]. This suggests that occupations' positions on the people-things dimension may predict their degree of sex segregation, but occupations' positions on the data-ideas dimension may not.

Identifying occupational characteristics that predict occupational sex segregation is complicated by the fact that sex segregation has changed over time as societal gender roles have changed [19]–[20]. Some U.S. occupations that were strongly male-dominated in the past are much less so today (e.g., lawyer, physician), with some shifting so dramatically that they are now female-dominated (e.g., accountant and auditor, psychologist). Still other occupations that were strongly male-dominated in the past continue to be so today (e.g., chemist, electrical engineer). Thus, when investigating what factors predict occupational sex segregation, researchers may benefit from taking a historical perspective. By studying occupational segregation over time, researchers can not only address the question—“What occupational characteristics predict occupational sex segregation?”—but they can also explore whether the answer to this question has changed over time.

The research reported here took such a historical perspective by analyzing U.S. Bureau of Labor Statistics data for women's rate of participation in 60 varied occupations from 1972 to 2010. In analyzing these statistics, we focused on the following questions: Are occupations' positions on the people-things and data-ideas dimensions related to their degree of sex segregation, and have these associations changed over time? Is the vertical dimension of occupational status also related to occupational sex segregation, and has the association between job status and sex segregation also changed over time? Finally, if we regard the three fundamental occupational characteristics identified here—occupational status, people-things orientation, and data-ideas orientation—as factors that predict the percent of women working in various jobs, then has the power of these three factors to predict occupational sex segregation changed over time?

## Methods

The portion of the study that made use of student ratings of occupational characteristics used data collected from college students and was approved by the Institutional Review Board (IRB) of California State University, Fullerton. Data were collected via an online survey in which participants received, as the first page of the survey, a consent statement that informed them of the nature of the study, the kinds of questions they would be asked, and that participation was anonymous and voluntary. The consent statement also included the following sentence: "All data/records will be kept confidential to the extent allowed by law" (inclusion of this statement was an IRB requirement). This consent procedure was approved by the IRB. Participants indicated that they had read the consent statement and wished to proceed with the online survey by continuing with the survey. Given that data were collected via an online survey, participants could cease participation at any point simply by closing the survey window in the browser of their computer.

U. S. Bureau of Labor Statistics data were compiled for the percent of women working in 60 occupations from 1972 to 2010. We selected occupations that met the following criteria: 1) Occupations had to be varied, representing varied status levels and all six categories in Holland's influential RIASEC model of vocational preferences and work settings—i.e., realistic, investigative, artistic, social, enterprising, and conventional occupations [21]. 2) A substantial number of workers had to be employed in each occupation over the time period studied. 3) Occupations had to be clearly and consistently represented in U. S. Bureau of Labor Statistics data over the time period studied, despite changes that occurred in occupational classification systems. Table 1 lists 60 occupations we compiled that met these criteria. Over the time period studied, workers in these occupations comprised about a third of the total U.S. workforce. A small number of data values were missing for some occupations, typically because the number of women working in an occupation was extremely low. In such cases, values were interpolated from corresponding values from adjacent years in non-MLM analyses.

**Table 1:** Sixty occupations ranked in order of status, people-things orientation, and data-ideas orientation scores.

Occupations ranked by status (from high to low status)	Occupations ranked by people-things orientation (from things-oriented to people-oriented)	Occupations ranked by data-ideas orientation (from data-oriented to ideas-oriented)
Physicians	Machinists	Property managers
Dentists	Aeronautical and astronautic engineers	Bank tellers
Lawyers	Chemists and materials scientists	Receptionists
Aeronautical and astronautic engineers	Automobile mechanics	Real estate agents and brokers
Pharmacists	Computer programmers	Secretaries
Bank officials or financial managers	Electrical and electronics engineers	Bank officials or financial managers
Civil engineers	Welders and flame cutters	Accountant and auditors
Airplane pilots	Computer systems analysts	Stock and bond sales agents/Securities & financial services sales
Chemists and materials scientists	Mechanical engineers	File clerks
Economists	Electricians	Postal clerks
Architects	Truck drivers	Cashiers
Electrical and electronics engineers	Civil engineers	Bookkeepers
Mechanical engineers	Drafters	Farmers
Industrial engineers	Roofers and slaters	Mail carriers, post office
Psychologists	Biological scientists	Waiters
Biological scientists	Airplane pilots	Lawyers
Computer systems analysts	Statisticians	Cooks
Stock and bond sales agents/Securities & financial services sales	Carpenters	Private household service occupations
Computer programmers	Farmers	Bus drivers
Statisticians	Painters, construction and maintenance	Police and detectives
Police and detectives	Industrial engineers	Airplane pilots
Registered nurses	Pharmacists	Truck drivers
Accountant and auditors	Clinical Laboratory technologists and technicians	Industrial engineers
Fire fighters	Architects	Hairdressers and cosmetologists
Drafters	Painters and sculptors	Fire fighters
Real estate agents and brokers	Accountant and auditors	Welders and flame cutters
Clinical Laboratory technologists and technicians	File clerks	Automobile mechanics
Musicians and composers	Cooks	Painters, construction and maintenance
Dietitians	Mail carriers, post office	Carpenters
Property managers	Economists	Roofers and slaters
Electricians	Postal clerks	Pharmacists
Editors and reporters	Bookkeepers	Electricians
Secondary school teachers	Fire fighters	Social workers
Social workers	Dentists	Clergy
Machinists	Bus drivers	Computer systems analysts
Elementary school teachers	Musicians and composers	Machinists
Clergy	Cashiers	Economists
Painters and sculptors	Private household service occupations	Statisticians
Postal clerks	Physicians	Dietitians
Carpenters	Bank tellers	Clinical Laboratory technologists and technicians
Librarians	Photographers	Drafters
Farmers	Property managers	Mechanical engineers
Mail carriers, post office	Stock and bond sales agents/Securities & financial services sales	Librarians
Automobile mechanics	Dietitians	Editors and reporters
Welders and flame cutters	Secretaries	Computer programmers
Photographers	Librarians	Civil engineers
Secretaries	Police and detectives	Physicians
Bank tellers	Bank officials or financial managers	Secondary school teachers
Painters, construction and maintenance	Registered nurses	Electrical and electronics engineers
Cooks	Lawyers	Registered nurses
Bookkeepers	Psychologists	Elementary school teachers
Roofers and slaters	Real estate agents and brokers	Photographers
Truck drivers	Editors and reporters	Aeronautical and astronautic engineers
File clerks	Receptionists	Dentists
Hairdressers and cosmetologists	Waiters	Musicians and composers
Receptionists	Hairdressers and cosmetologists	Chemists and materials scientists
Bus drivers	Secondary school teachers	Architects
Waiters	Elementary school teachers	Painters and sculptors
Cashiers	Social workers	Biological scientists
Private household service occupations	Clergy	Psychologists

Occupations' status levels were assessed via: (1) statistics for occupations' median incomes and (2) student ratings of occupations' status and income levels. Median income statistics were obtained from O*NET OnLine, the U. S. Department of Labor's Occupation Information Network (www.onetonline.org). In some cases, median incomes for several subordinate occupations (e.g., several kinds of psychologists) were averaged to provide a median income for the superordinate occupational category we used (e.g., “psychologist”). To obtain subjective ratings of occupational status, we asked 78 college students to rate each occupation on “income (expected salary)” using a 5-point scale that ranged from “very low income” to “very high income” and on “social status and prestige” using a 5-point scale that ranged from “very low status” to “very high status.” With occupations serving as the unit of analysis, mean student ratings of income and status were highly reliable (coefficient alpha  = .99 for both), and median incomes correlated strongly with mean student ratings of occupations' income levels (*r* = .80, *p*<.001) and with mean student ratings of occupations' status levels (*r* = .77, *p*<.001).

Occupations' positions on the people-things and ideas-data dimensions were assessed from O*NET statistics. A National Center for O*NET Development publication ([22], Appendix B) provided ratings, made by three expert raters, of how well occupations were described by the six kinds of work environments identified by Holland's hexagon model: realistic, investigative, artistic, social, enterprising, and conventional. These ratings displayed high inter-rater reliability [22]. In some cases, work environment ratings for several subordinate occupations were averaged to provide work environment ratings for the superordinate occupational category we used. For each occupation, we averaged the three expert ratings for a given RIASEC dimension and then used these mean ratings to compute people-things and ideas-data scores, using the following empirically derived formulas discussed in Su, Rounds, and Armstrong [18] and provided to us by R. Su: People-things  = 2× Realistic + Investigative – Artistic –2× Social – Enterprising + Conventional; and Data-Ideas  = −1.73× Investigative −1.73× Artistic +1.73× Enterprising +1.73× Conventional.

To obtain a second measure of occupations' people-things orientation, we asked 78 college students to rate each occupation on a 5-point scale that ranged from “very people-oriented” to “very things-oriented.” Rating instructions asked participants to rate how much a job dealt “with ‘people’ (e.g., managing, thinking about, and counseling people) versus…with ‘things’ (dealing with and thinking about nonhuman things such as machines, computers, mathematics, and mechanisms).” With occupations serving as the unit of analysis, students' mean people-things ratings were highly reliable (coefficient alpha  = .99) and correlated strongly with the people-things scores computed from O*NET expert ratings, *r* = .83, *p*<.001.

To explore the relation between occupational characteristics and the percent of women employed in the 60 assessed occupations over time, we conducted multilevel linear modeling (HLM) analyses, with occupations serving as the units of analysis. We conducted two MLM analyses, one using the O*NET measures of occupational characteristics (i.e., median income, and people-things and ideas-data scores computed from expert ratings) and the other using mean student ratings of jobs' status and people-things orientation as predictors. We regarded the first set of predictors as comprising more objective measures and the second set as comprising more subjective measures of occupational characteristics.

## Results

### Broad Patterns in the Data

To generate composite measures of occupational status and people-things orientation we averaged student ratings of job status and income (to form a two-item student-rating scale of job status) and then computed Z scores for this variable as well as for median job income, student ratings of people-things orientation, and O*NET-based people-things scores. The two Z-scored status measures were then averaged to form a composite status measure, and the two Z-scored people-things measures were averaged to form a composite people-things measure. Regarded at two-item scales, these measures were highly reliable (coefficient alpha  = .89 for composite status measure and .91 for composite people-things measure). Table 1 lists the 60 occupations ranked by status, people-things orientation, and data-ideas orientation, as assessed by composite status and people-things scores and by O*NET-based ideas-data scores.

**Figure 1:**
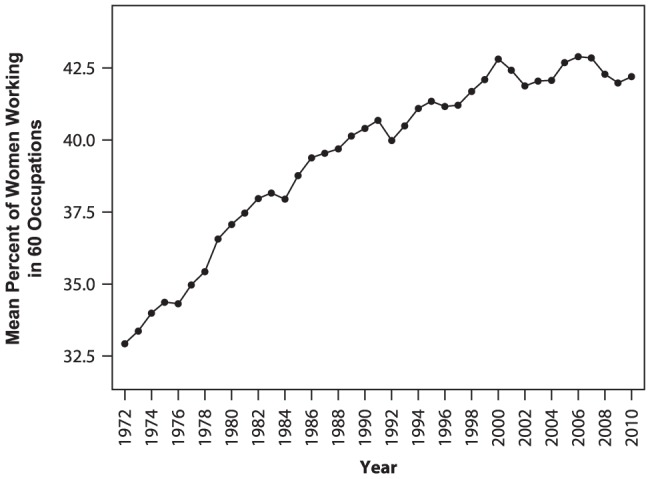
Mean percent of women working in 60 occupations as a function of year.

To provide an initial assessment of patterns in women's participation in occupations over time, we conducted a one-way repeated-measures ANOVA on the percent of women working in occupations, with year serving as the repeated measures factor and composite measures of occupational status and people-things and O*NET-based data-ideas serving as covariates. Occupations served at the units of analysis. Tests of within-subjects effects (in our case, within-occupations effects) showed a main effect for years, *F*(38, 2128) = 27.75, *p*<.001, partial *η^2^* = .33, with women's average participation in occupations increasing over time (see Figure 1). There was also a significant interaction between year and status, *F*(38, 2128)  = 19.66, *p*<.001, partial *η^2^* = .26, which reflected the fact that women's rates of participation particularly increased over time for high-status jobs (a finding further explored in MLM analyses reported later). Between-subjects (i.e., between occupations) effects showed that both occupational status (*F*(1, 56)  = 9.11, *p* = .004, partial *η^2^* = .14) and people-things scores ((*F*(1, 56)  = 66.54, *p*<.001, partial *η^2^* = .35) were significantly associated with the percent of women working in occupations, but data-ideas scores were not ((*F*(1, 56)  = .07).

Simple correlations showed that people-things orientation correlated −.61 (*p*<.001) with the mean percent of women working in occupations over 39 years, with fewer women working in things-oriented than in people-oriented occupations. Occupational status correlated −.42 (*p* = .001) with the mean percent of women working in occupations, with fewer women working in high than low status jobs. Finally, data-ideas scores correlated .27 (*p*<.05) with the mean percent of women working in occupations, with fewer women working in ideas-oriented than in data-oriented jobs. These effect sizes ranged from “medium” to “large.”

Across occupations, ideas-data orientation was significantly correlated with status, *r* = −.41, *p*<.01, whereas people-things orientation was not significantly correlated with status or ideas-data (respective *r*s = .14 and −.21). A regression predicting mean percent of women working in occupations from all three occupational characteristics yielded a multiple *R* of .69, *p*<.001, with significant β weights for people-things (β = −.55, *p*<.001) and status (β = −.32, *p*<.01), but not for ideas-data (β = .03), suggesting that ideas-data did not predict women's participation in occupations when its overlap with status was controlled for.

### Multilevel Modeling Analyses

With occupations serving as the units of analysis, we used the PROC MIXED procedure of SAS [23] to explore: 1) how women's levels of participation in individual occupations varied across occupations and whether the time trajectories of women's participation in occupations varied across occupations, and 2) whether occupational characteristics (status, people-things orientation, and data-ideas orientation) predicted the initial level of women's representation in occupations and the rate of change (slope) of women's participation in various occupations over time. MLM analyses were deemed appropriate because time was nested within occupations.

MLM has two levels of analysis and estimation. In the current analyses, the first level estimated the growth trajectories of women's participation rates for each occupation. Each occupation's growth trajectory was described by an intercept (the level of women's participation in 1972) and a slope (the linear rate of change in the percent of women in occupations over 39 years). The second level of analysis investigated whether intercepts and time slopes for the 60 occupations varied as a function of occupational characteristics.

An autoregressive error structure with lag of one was imposed on the error covariance structure [24], based on the assumption that the percent of women in an occupation was more similar in adjacent years than distal years. A logit transformation was performed on the percent of women in an occupation during a given year because percent measures were bounded variables, which violated an MLM assumption that the dependent variable be continuous and unbounded.

#### Model 1: Median income and O*NET people-things and data-ideas scores as occupational characteristics

The first MLM analysis assessed the effects of three O*NET-based occupational characteristics on the percent of women working in the 60 occupations over 39 years. The first-level unit of analysis was the percent of women from 1972 to 2010 in each of the 60 occupations. Second-level units of analysis were the 60 occupations and the three assessed occupational characteristics. Random effects for the effect of occupations were included in the model to assess the variability in the percent of women in occupations and in the linear growth rates of these percents over time as a function of the three occupational characteristics. The model was specified as follows:

L1: 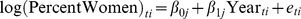


L2: 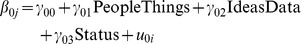


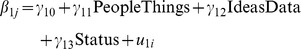



Model 1 results are presented in Table 2. The linear growth rates of women's participation in occupations from 1972 to 2010 varied significantly across occupations (random effect 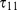). The significant residual of .068 indicated that a significant amount of variability in the percent of women in occupations was left unexplained by the model.

**Table 2:** Fixed and random effects of Model 1, which predicted the change in percent of women in an occupation from O*NET-based measures of occupations' people-things orientation, data-ideas orientation, and status.

Effects	Estimate	*e^b^*	S.E.
Fixed			
Intercept	0.776**	2.172	0.1193
Year	−0.014	0.986	0.017
People-Things	−0.122**	0.885	0.007
Ideas-Data	0.042**	1.043	0.008
Status	−2.36**	0.094	0.174
People-Things*Year	0.001	1.001	0.001
Ideas-Data*Year	−0.001	0.999	0.001
Status*Year	0.052*	1.052	0.025
Random			
*τ_11_*	0.003**		0.001
*e_ti_*	0.68**		0.029

*p<.05,

**p<.01.

**Figure 2:**
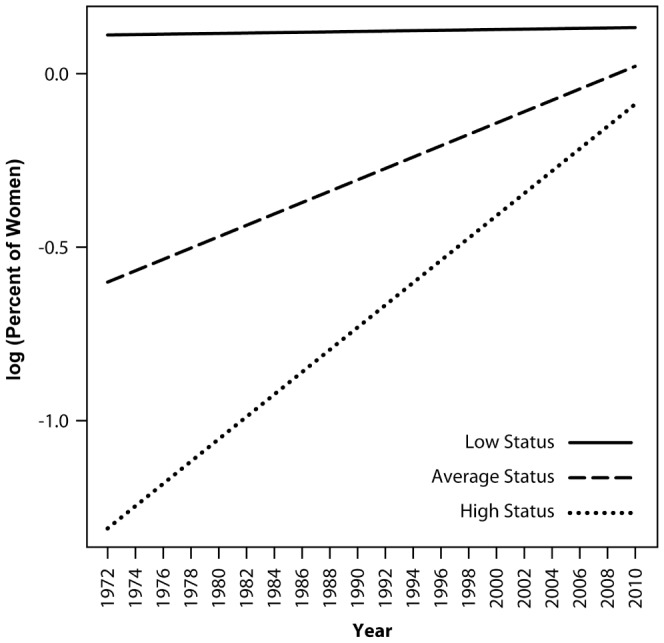
Simple slope plots of percent of women in low, average, and high-status occupations in MLM Model 1. Low-status occupations were defined as one SD below the mean, average-status occupations as at the mean, and high-status occupations as one SD above the mean status level of all occupations. Status was defined in terms of occupations' median income levels.

Results for fixed effects in Table 2 showed a nonsignificant effect for year. This counter-intuitive finding resulted from the fact that the previously described increase in women's overall participation in occupations from 1972 to 2010 was absorbed into a significant status by year interaction, which is described later. There was a significant fixed effect for people-things, with more women working in people-oriented than things-oriented occupations. There was no significant interaction between people-things and year—i.e., occupations' people-thing scores did not predict changes over time in women's participation in occupations. Occupations' data-ideas scores were related to women's participation rates in occupations, with more women in data-oriented than in ideas-oriented occupations. Finally, occupations' median income predicted the percent of women working in occupations, with more women working in low-status than in high-status jobs. Furthermore, the interaction between status and year was significant, which reflected the tendency for women's representation to increase most dramatically from 1972 to 2010 in higher status jobs (see Fig. 2, which presents simple slope plots of increases in women's participation rates in low status, medium status, and high status occupations).

#### Model 2: Student ratings of job status and people-things orientation as occupational characteristics

Model 2 used student ratings of occupational status and income (averaged into a single “status” measure) and student ratings of occupations' people-things orientation as measures of occupational characteristics. Data-ideas orientation was excluded as a predictor because it was not assessed via student ratings. The first-level and second-level units of analysis corresponded to those described in Model 1. Model 2 was specified as follows:

L1: 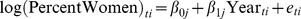


L2: 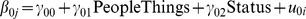


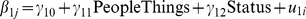



**Figure 3:**
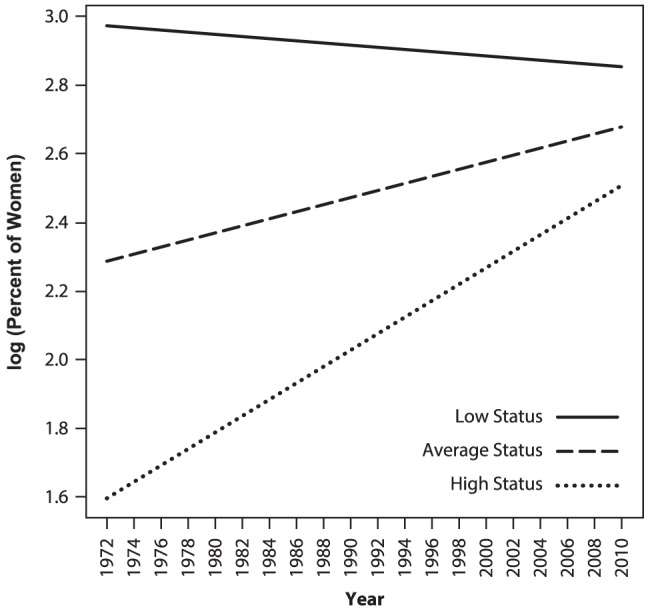
Simple slope plots of percent of women in low, average, and high-status occupations in MLM Model 2. Low-status occupations were defined as one SD below the mean, average-status occupations as at the mean, and high-status occupations as one SD above the mean status level of all occupations. Status was defined in terms of mean student ratings of occupations' income and status levels.

Model 2 results are presented in Table 3. As was true for Model 1, random effects again indicated significant variability in linear growth rates and significant variability left unexplained by the model. Model 2 yielded a nonsignificant effect for year. As in Model 1, this counter-intuitive finding resulted from the fact that the previously described increase in women's overall participation in occupations from 1972 to 2010 was absorbed into a significant status by year interaction, which is described later. There was a significant fixed effect for occupations' people-things orientation, with more women working in people-oriented than thing-oriented jobs. Again, there was not a significant interaction between people-things and year. Student-rated occupational status was related to the percent of women working in occupations in 1972, with more women found in low status than high status occupations. Finally, the interaction between status and year was again significant, reflecting the fact that women's participation increased most dramatically over time in higher status occupations (see Fig. 3, which presents simple slope plots of increases in women's participation rates in low status, medium status, and high status occupations).

**Table 3:** Fixed and random effects of Model 2, which predicted the change in percent of women in an occupation from student ratings of occupations' people-things orientation and status.

Effects	Estimate	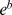	S.E.
Fixed			
Intercept	5.169**	164.84	0.226
Year	−0.046	0.957	0.031
People-Things	−1.062**	0.342	0.047
Status	−0.944**	0.403	0.065
People-Thing*Year	0.002	1.002	0.007
Status*Year	0.019**	1.017	0.009
Random			
*τ_11_*	0.002**		0.0004
*e_ti_*	0.611**		0.0263

*p<.05,

**p<.01.

### Year-by-year regressions predicting women's participation in occupations from occupational characteristics

Given that MLM analyses showed that increasing numbers of women entered high-status jobs from 1972 to 2010 but, simultaneously, women's participation in things-oriented jobs tended to remain low and stable over the same time, we posed a final empirical question: Did the relative power of occupational status and people-things orientation to predict women's participation in occupations change from 1972 to 2010? To address this question, we conducted 39 regressions—one for each assessed year—that predicted the percent of women working in assessed occupations from the composite scores for occupational status and people-things orientation and O*NET-based data-ideas scores. Each regression yielded β weights for status, people-things orientation, and data-ideas orientation. All regressions were significant and accounted for a substantial amount of variance, with multiple *R* values ranging from .66 to .70. The β weights for both status and people-things were significant in all 39 regressions, whereas β weights for data-ideas were not significant in all regressions.

**Figure 4:**
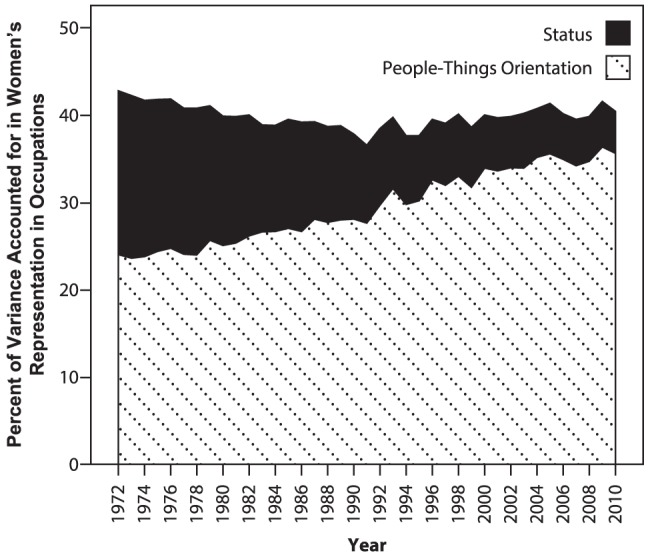
Amount of variance in the percent of women working in occupations accounted for by occupations' status and people-things orientation for each year from 1972 to 2010.

Because occupational status and people-things were only weakly correlated (*r* = .14, *ns*), β weights were converted to β^2^ (amount of unique variance accounted for by each predictor). Figure 4 graphically portrays the amount of unique variance accounted for by job status and people-things orientation for each year from 1972 to 2010. People-things orientation accounted for slightly more variance than status did in 1972 (24 versus 19 percent, respectively). However, by 2010 people-things accounted for more than seven times as much variance as occupational status did (36 percent versus 5 percent, respectively). Thus, as women increasingly entered high-status occupations from 1972 to 2010, job status became an increasingly weak predictor of women's participation in occupations, while occupations' people-things orientation became an increasingly strong predictor.

## Discussion

Our findings provide new insights into the empirical puzzles in occupational sex segregation research noted by various researchers [3], [11], [12], [13]. First, they confirm that the current link between job status and occupational sex segregation in the U.S. is relatively weak, but they also show that this link was stronger in the past. As occupational status has become a less powerful predictor of women's participation in occupations over time, other factors—such as occupations' people-things orientation—have become stronger predictors.

The second empirical puzzle—that occupational sex segregation tends to be stronger in economically developed, gender egalitarian countries than in less developed, more gender traditional countries—was not directly addressed by our study. However, our results suggest new ways of thinking about such cross-national findings. If other economically developed countries are similar to the United States, then occupational status has become an increasingly weak predictor of occupational sex segregation in these countries too, while occupations' people-things orientation has become an increasingly strong predictor. We hypothesize that as the restrictions of traditional gender roles weakened in economically developed nations in recent decades, women who possessed the requisite human capital increasingly pursued and entered high-status occupations. However, women were simultaneously freer to express their interests and values through their occupational choices, and one consequence may have been that women showed a marked preference for high-status jobs that were people-oriented rather than things-oriented. We hypothesize that, in contrast, many women (and men) in economically undeveloped countries do not have the luxury of pursuing work based on their interests but rather must accept whatever jobs are available, and this may have the effect of reducing some kinds of occupational sex segregation in these countries.

Finally, the current findings provide an explanation for the third empirical puzzle identified by researchers—that changes in occupational sex segregation have been slow to occur and uneven across occupations. As shown by our analyses, links between job status and occupational sex segregation in the United States have weakened considerably over the past 40 years as women have increasingly entered a variety of high-status occupations. However, simultaneously, women's representation in things-oriented jobs—regardless of jobs' status levels—has remained low (for example, in 2010 women comprised, on average, only 15 percent of the workers in the 20 most things-oriented jobs in our list, whereas they comprised 62 percent of workers in the 20 most people-oriented jobs). Thus, one factor—job status—has led to a reduction in occupational sex segregation over the past 40 years (i.e., increasing numbers of women have entered many formerly male-dominated high-status occupations), whereas another factor—jobs' people-things orientation—has served to maintain occupational sex segregation (women continue to be found much more in people-oriented than in things-oriented occupations at all job status levels).

The current results may inform discussions of how to increase women's representation in occupations that remain male-dominated. For example, our results suggest that in addition to posing the question—Why do women sometimes work in lower status jobs than men?—researchers and policy makers should increasingly address the question: Why do women, on average, pursue different kinds of occupations than men do at all job status levels? Given that occupations' people-things orientation has become an increasingly potent predictor of women's participation in occupations over the past 40 years, future research should address two applied questions as well: How malleable are women's and men's preferences for people-oriented and things-oriented jobs, and can sex differences in preferences for people-oriented and things-oriented jobs be reduced through educational and social interventions?
